# Supplementary figures and images for: Validation of the Vietnamese version of the Montgomery-Asberg depression rating scale (MADRS) in rheumatoid arthritis patients
Source: PLOS Ment Health. 2025 Jul 7;2(7):e0000277. doi: 10.1371/journal.pmen.0000277 (PMC12798514; doi:10.1371/journal.pmen.0000277)

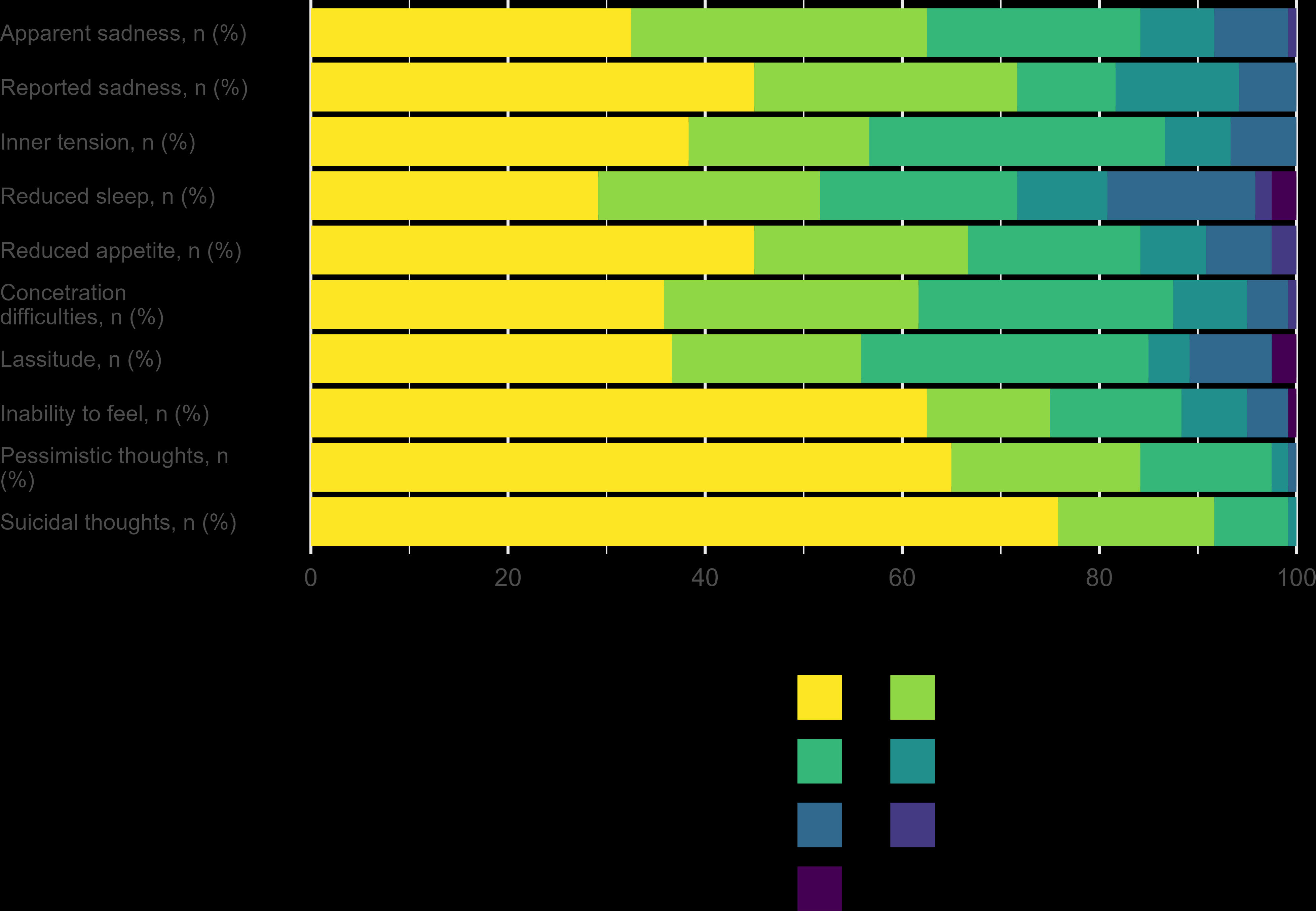

Supplement: S1 Fig — (TIF) [file pmen.0000277.s001.tif]

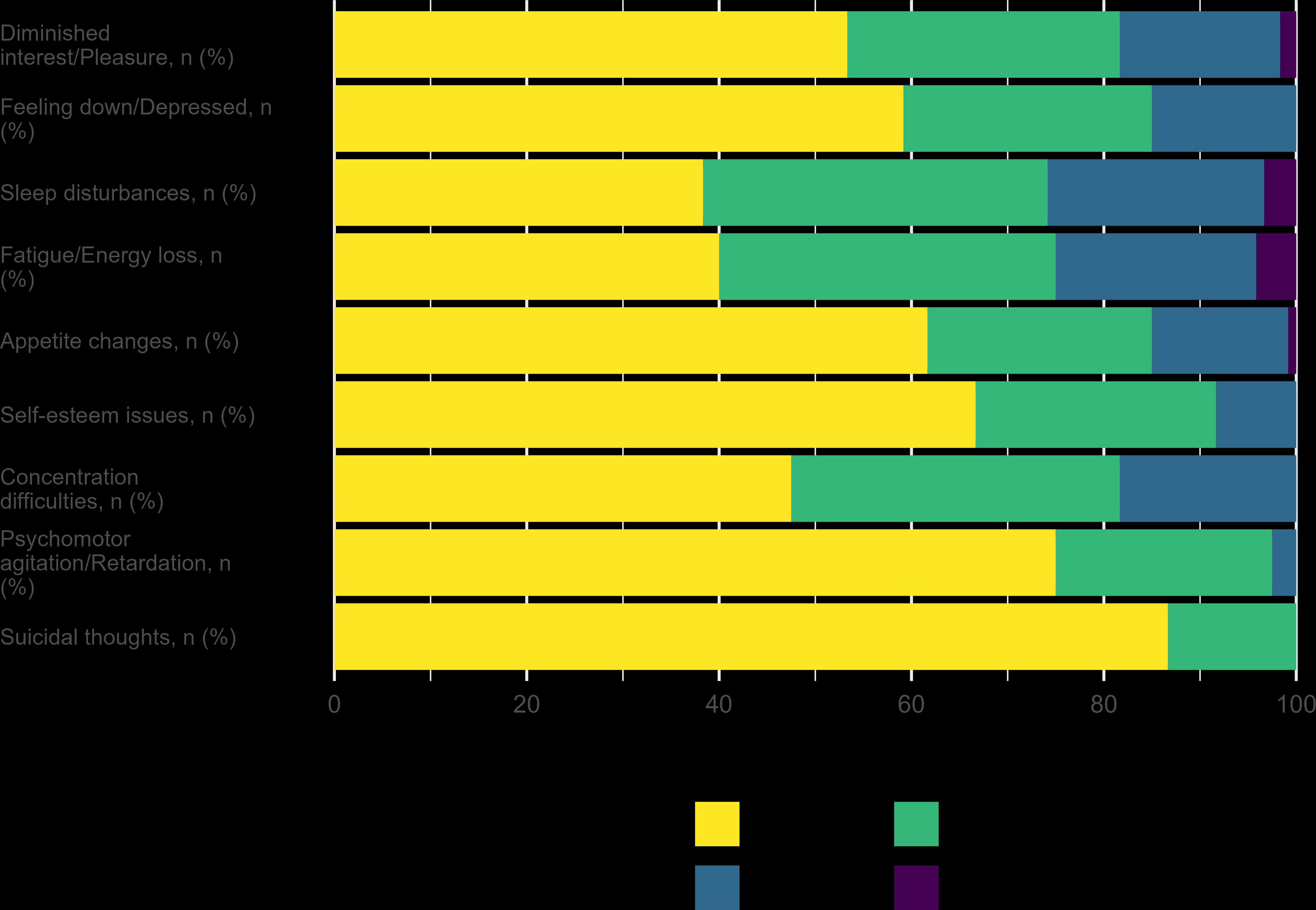

Supplement: S2 Fig — (TIF) [file pmen.0000277.s002.tif]

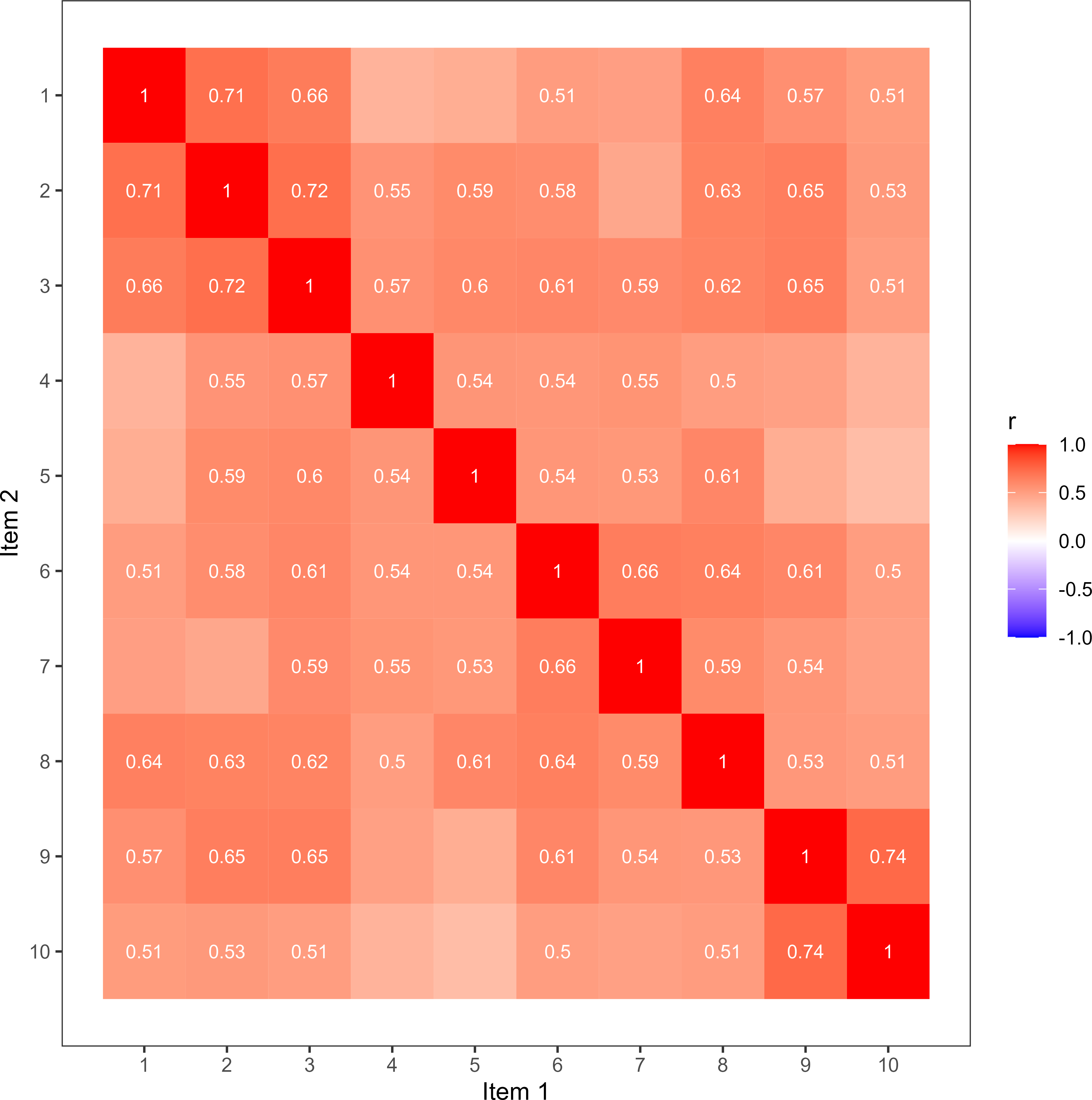

Supplement: S3 Fig — (TIF) [file pmen.0000277.s003.tif]

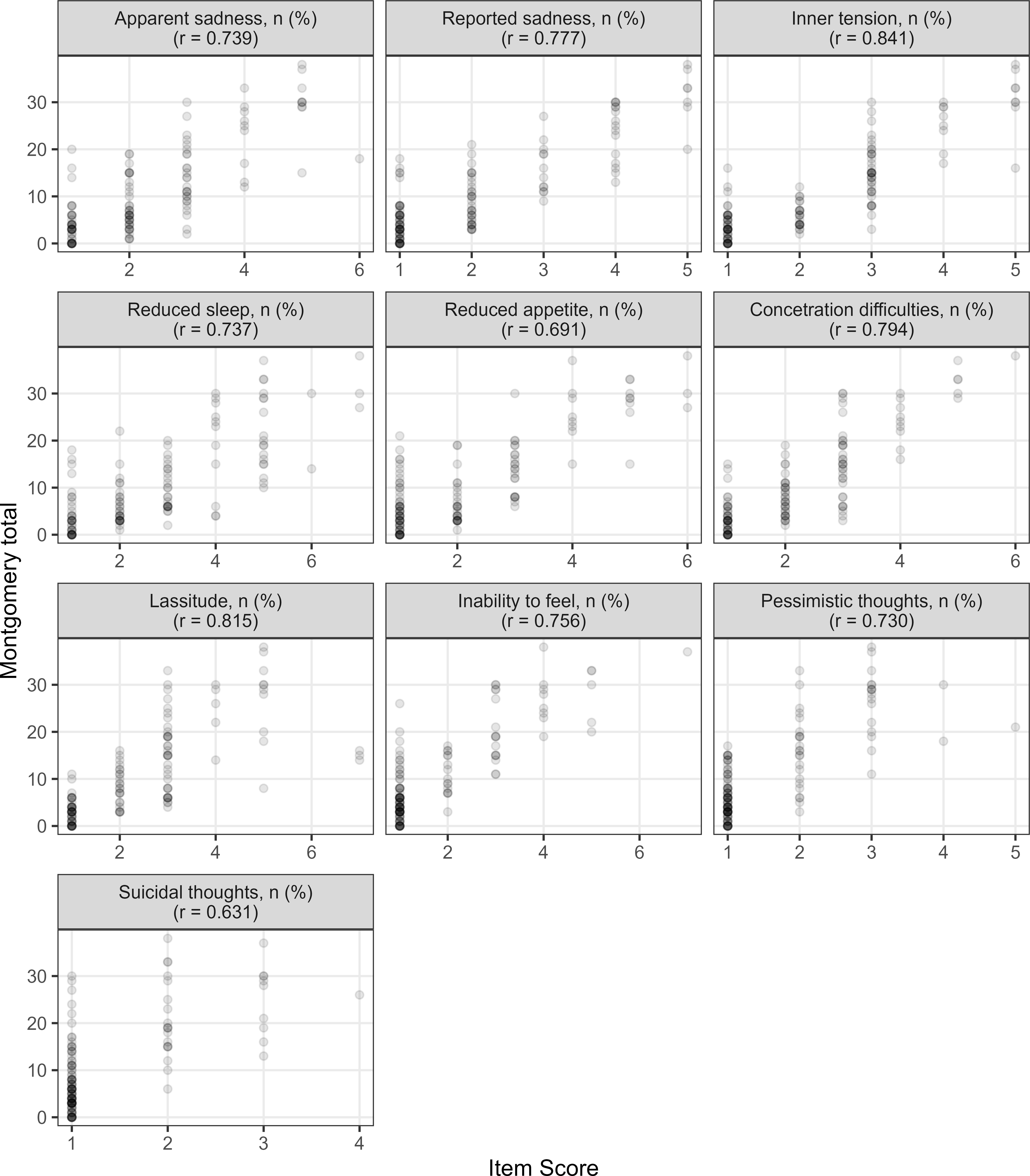

Supplement: S4 Fig — (TIF) [file pmen.0000277.s004.tif]

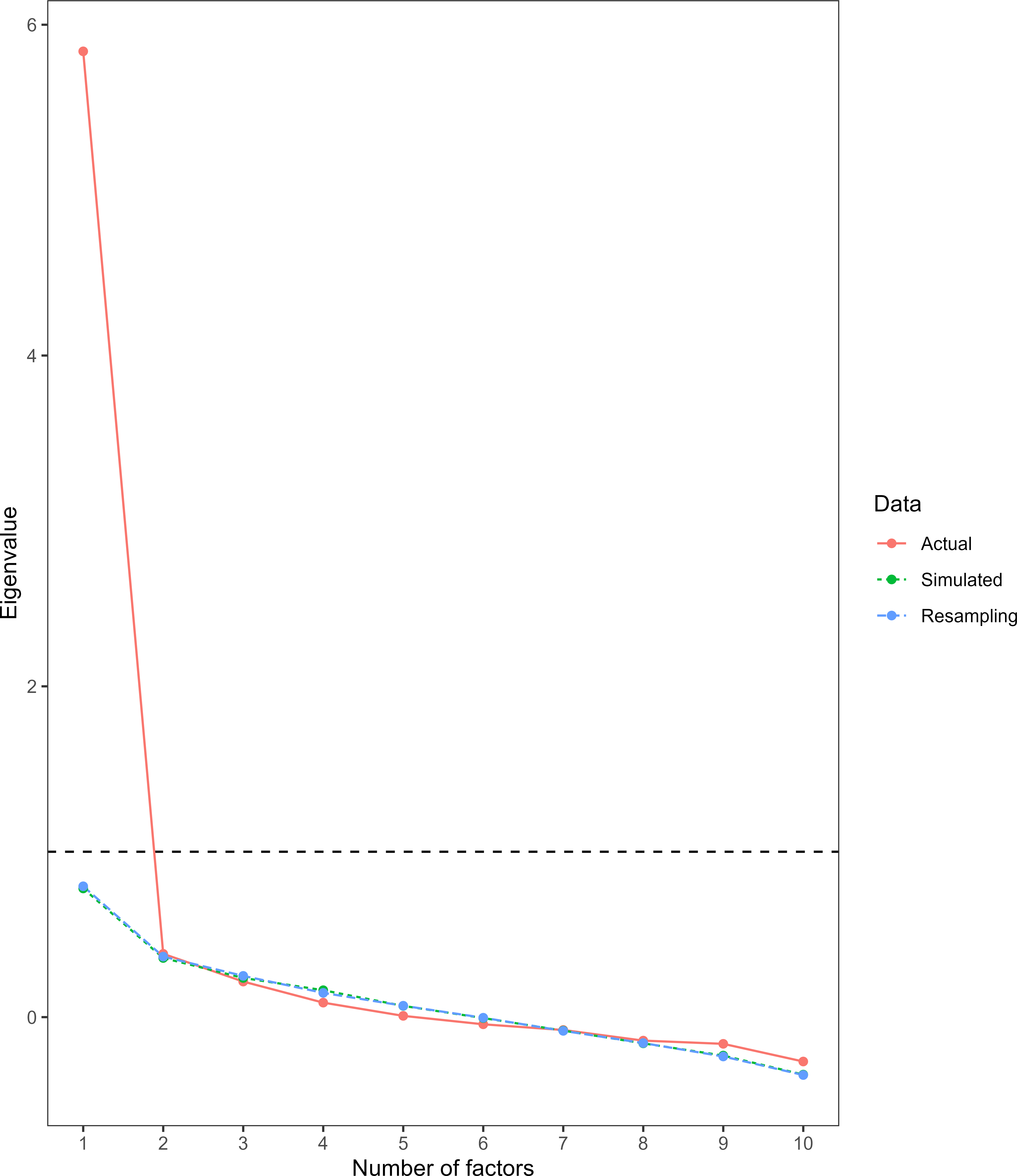

Supplement: S5 Fig — (TIF) [file pmen.0000277.s005.tif]

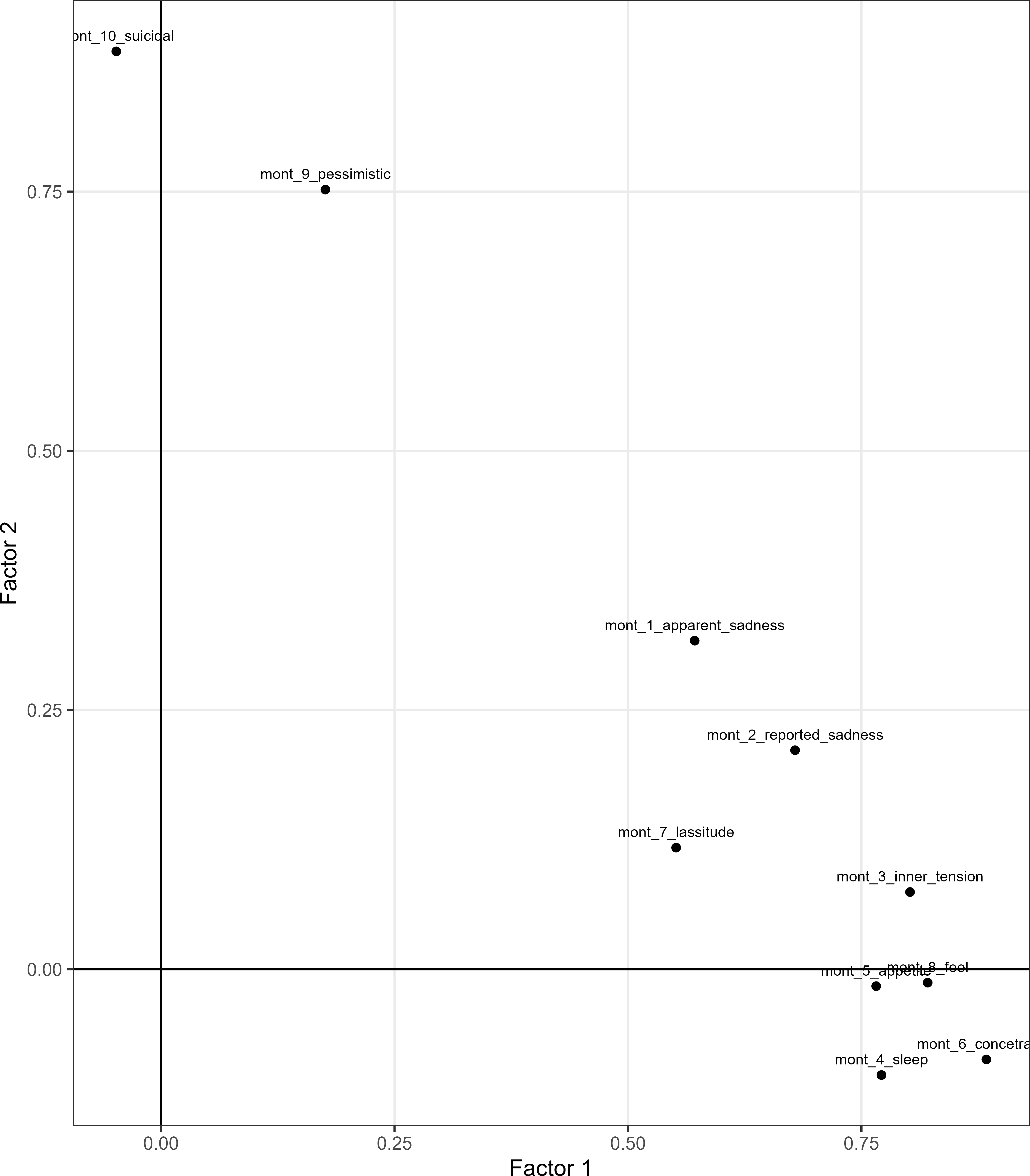

Supplement: S6 Fig — (TIF) [file pmen.0000277.s006.tif]

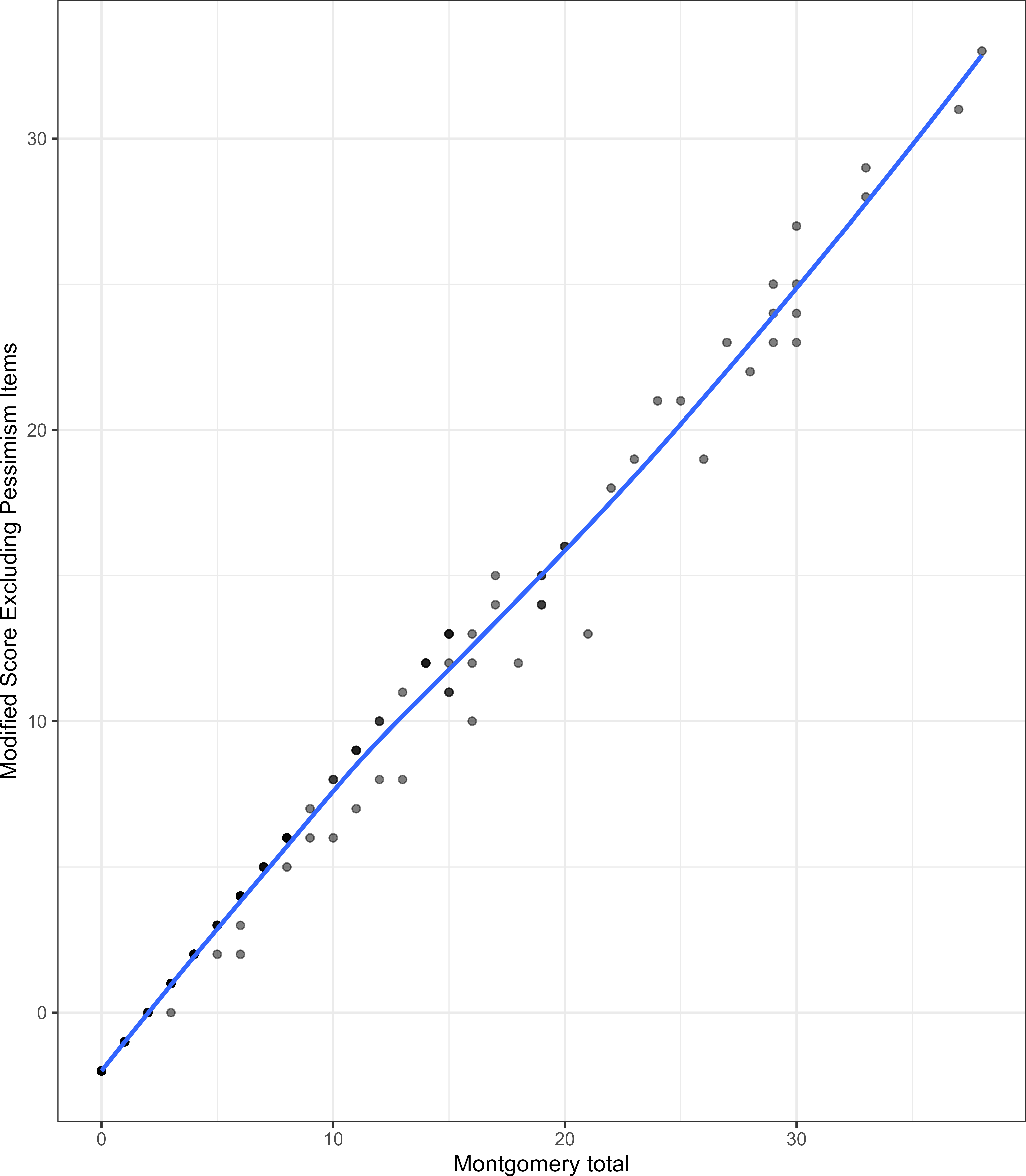

Supplement: S7 Fig — (TIF) [file pmen.0000277.s007.tif]
